# Pauci-Immune Crescentic Glomerulonephritis Associated With Primary Sjögren Syndrome: A Case Report

**DOI:** 10.7759/cureus.102261

**Published:** 2026-01-25

**Authors:** Fabian M Rivas Vega, María F Mendoza Rodriguez, Jaime Loeza-Suárez

**Affiliations:** 1 Internal Medicine, Hospital Juárez de México, Mexico City, MEX; 2 Internal Medicine, Hospital Juárez de México, México City, MEX

**Keywords:** anca negative, pauci-immune crescentic glomerulonephritis, primary sjögren's syndrome, rapidly progressive glomerulonephritis (rpgn), renal biopsy, renal involvement, sjögren syndrome

## Abstract

Primary Sjögren syndrome (SS) is a chronic systemic autoimmune disease predominantly characterized by exocrine gland involvement; however, extraglandular manifestations, including renal involvement, may occur and contribute significantly to morbidity. Renal involvement most commonly presents as tubulointerstitial nephritis, while glomerular disease is considerably less frequent. Pauci-immune crescentic glomerulonephritis (GN) is an exceptionally rare renal manifestation of primary SS and is typically associated with antineutrophil cytoplasmic antibody (ANCA)-related vasculitis, although antibody negativity has been described in isolated cases.

We report the case of a 30-year-old female with no significant past medical history who presented with hypertensive crisis and rapidly progressive renal dysfunction. Laboratory evaluation revealed acute kidney injury with significant proteinuria. Further evaluation identified sicca symptoms, positive antinuclear antibodies at high titers, and elevated anti-SS-related antigen A antibodies, with normal complement levels and negative ANCAs. Minor salivary gland biopsy confirmed the diagnosis of primary SS. Renal biopsy demonstrated pauci-immune crescentic GN with advanced chronic changes and absence of immune complex deposition on immunofluorescence. The patient was treated with intravenous pulse corticosteroids, resulting in improvement of renal function. This report highlights a rare and atypical form of renal involvement in primary SS and underscores the importance of considering pauci-immune GN in patients with SS presenting with rapidly progressive renal failure, even in the absence of ANCAs. Early recognition and prompt histopathological diagnosis are essential to guide appropriate immunosuppressive therapy and improve renal outcomes.

## Introduction

Sjögren syndrome (SS) is a chronic systemic autoimmune disease characterized by lymphocytic infiltration of the exocrine glands, particularly the salivary and lacrimal glands, leading to the classic symptoms of xerostomia and xerophthalmia [1-3]. This condition may present as primary SS, when it occurs in isolation, or as secondary SS, when associated with other systemic autoimmune diseases such as systemic lupus erythematosus or rheumatoid arthritis [4,5]. Beyond glandular involvement, SS may present with extraglandular manifestations that reflect its systemic nature. Among these, renal involvement represents a relevant cause of morbidity and may occur through different pathophysiological mechanisms. Tubulointerstitial nephritis is the most common renal manifestation, followed by immune complex-mediated glomerulonephritis (GN), particularly cryoglobulinemic GN [1]. Although these renal manifestations often respond to immunomodulatory therapies, early recognition and timely treatment are essential to prevent progression to advanced or end-stage chronic kidney disease [1-18].

Less common forms of glomerular involvement in SS, such as pauci-immune GN, represent a diagnostic challenge due to their low frequency and potentially aggressive clinical course. Pauci-immune GN is characterized by a rapid decline in renal function, usually over weeks to months, and by the presence of extensive glomerular crescents. From an immunopathological perspective, it is defined by the absence or paucity of immune deposits detected by immunofluorescence or electron microscopy [14,15,18]. We present the case of a patient with primary SS who developed rapidly progressive GN. The definitive diagnosis of pauci-immune GN was established by renal biopsy in the absence of antineutrophil cytoplasmic antibody (ANCA) positivity, highlighting a rare renal manifestation of this autoimmune disease.

## Case presentation

A 30-year-old female with no known chronic medical conditions presented to the emergency department with generalized malaise. Initial vital signs revealed a hypertensive crisis. During the initial evaluation, laboratory testing demonstrated elevated serum creatinine, prompting hospital admission for evaluation and management of acute kidney injury. During the first days of hospitalization, laboratory evaluation showed progressive worsening of renal function, with elevated serum creatinine and blood urea nitrogen, while liver function tests and serum albumin levels remained within normal limits, and serum electrolytes were also within reference ranges (Table 1). A 24-hour urine collection revealed significant proteinuria and reduced creatinine clearance, consistent with impaired renal function and proteinuric kidney disease (Table 1).

**Table 1:** Laboratory findings during hospitalization Laboratory parameters demonstrating acute kidney injury with significant proteinuria. Dashes indicate values not available at admission

Parameter	At admission	Peak value	Reference range
Serum creatinine (mg/dL)	3.2	4.23	0.6–1.2
Blood urea nitrogen (mg/dL)	—	51	7–20
Urea (mg/dL)	—	109	15–40
Serum albumin (g/dL)	3.8	3.8	3.5–5.0
Uric acid (mg/dL)	—	8.5	2.5–7.0
24-hour proteinuria (g/24 h)	—	2.23	<0.15
Creatinine clearance (mL/min)	—	17.12	>90
Sodium (mEq/L)	—	140	135–145
Potassium (mEq/L)	—	4.3	3.5–5.1
Calcium (mg/dL)	—	8.6	8.5–10.5
Phosphorus (mg/dL)	—	4.7	2.5–4.5

During hospitalization, directed history-taking and physical examination revealed symptoms of ocular and oral dryness. Based on these findings, an immunological workup was initiated. Antinuclear antibodies were positive at high titers, and anti-Sjögren syndrome-related antigen A antibodies were elevated. Anti-Smith antibodies were negative. ANCA testing was negative, including anti-myeloperoxidase and anti-proteinase 3 antibodies. Complement levels and immunoglobulin levels were within normal ranges (Table 2).

**Table 2:** Immunological and serological findings Immunological profile supporting the diagnosis of primary Sjögren syndrome and excluding ANCA-associated vasculitis SSA: Sjögren-syndrome-related antigen A; ANCA: antineutrophil cytoplasmic antibody; IgA: immunoglobulin A

Test	Result	Reference range
Antinuclear antibodies (ANA)	Positive (1:1000)	Negative
ANA pattern	Fine granular nuclear and reticular cytoplasmic	—
Anti-SSA (Ro) antibodies (UR/mL)	125.21	<20
Anti-Smith antibodies (UR/mL)	2.14	<20
ANCA	Negative	Negative
Anti-myeloperoxidase (UR/mL)	4.63	<20
Anti–proteinase 3 (UR/mL)	4.66	<20
Complement C3 (mg/dL)	93.2	90–180
Complement C4 (mg/dL)	17.1	10–40
IgA	Normal	Normal
IgE	Normal	Normal
IgG	Normal	Normal
IgM	Normal	Normal

**Figure 1:**
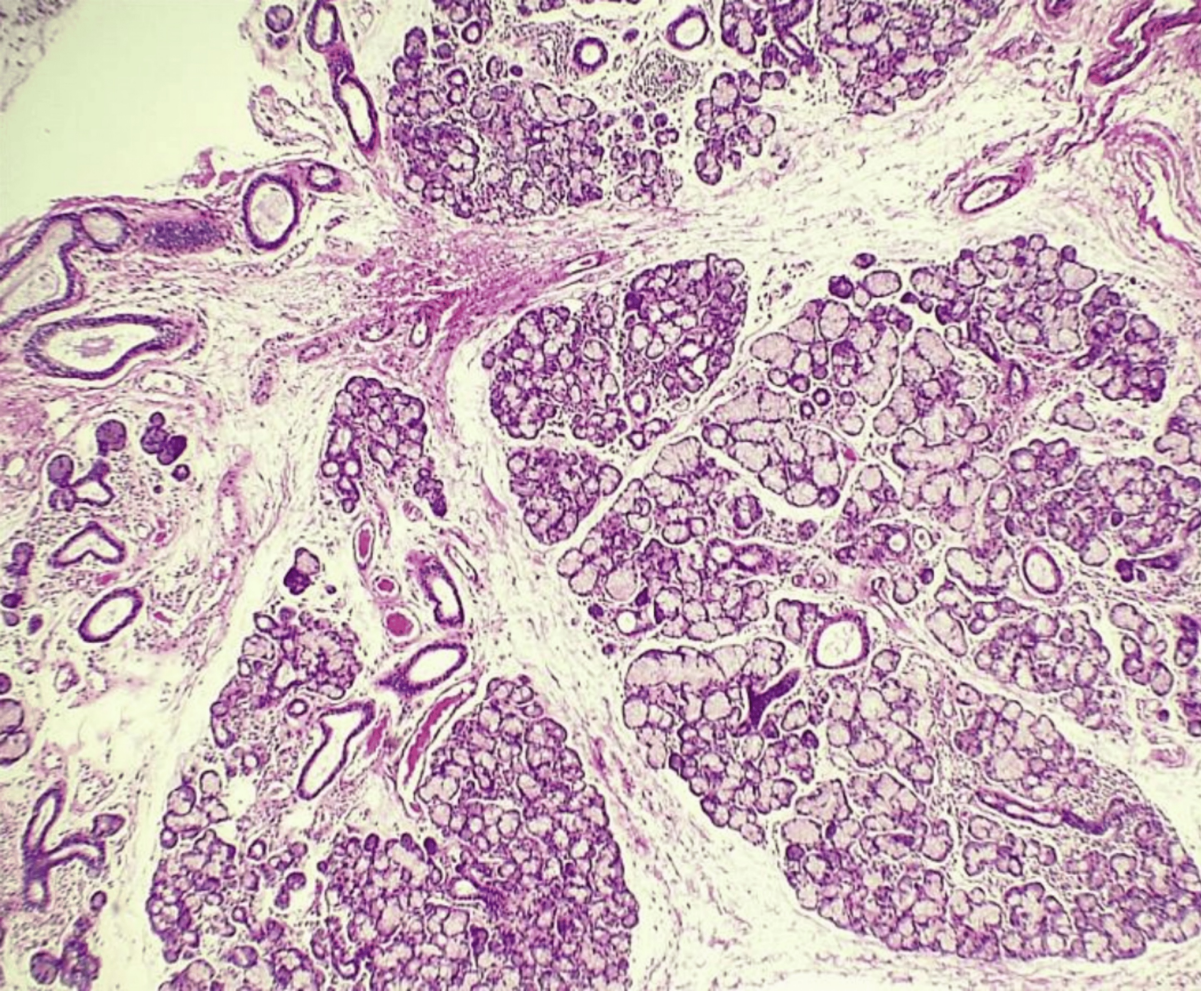
Minor salivary gland biopsy (hematoxylin and eosin stain, 4×) Low-power view showing preserved lobular architecture with multifocal lymphocytic infiltration and mild interstitial fibrosis within the minor salivary gland

**Figure 2:**
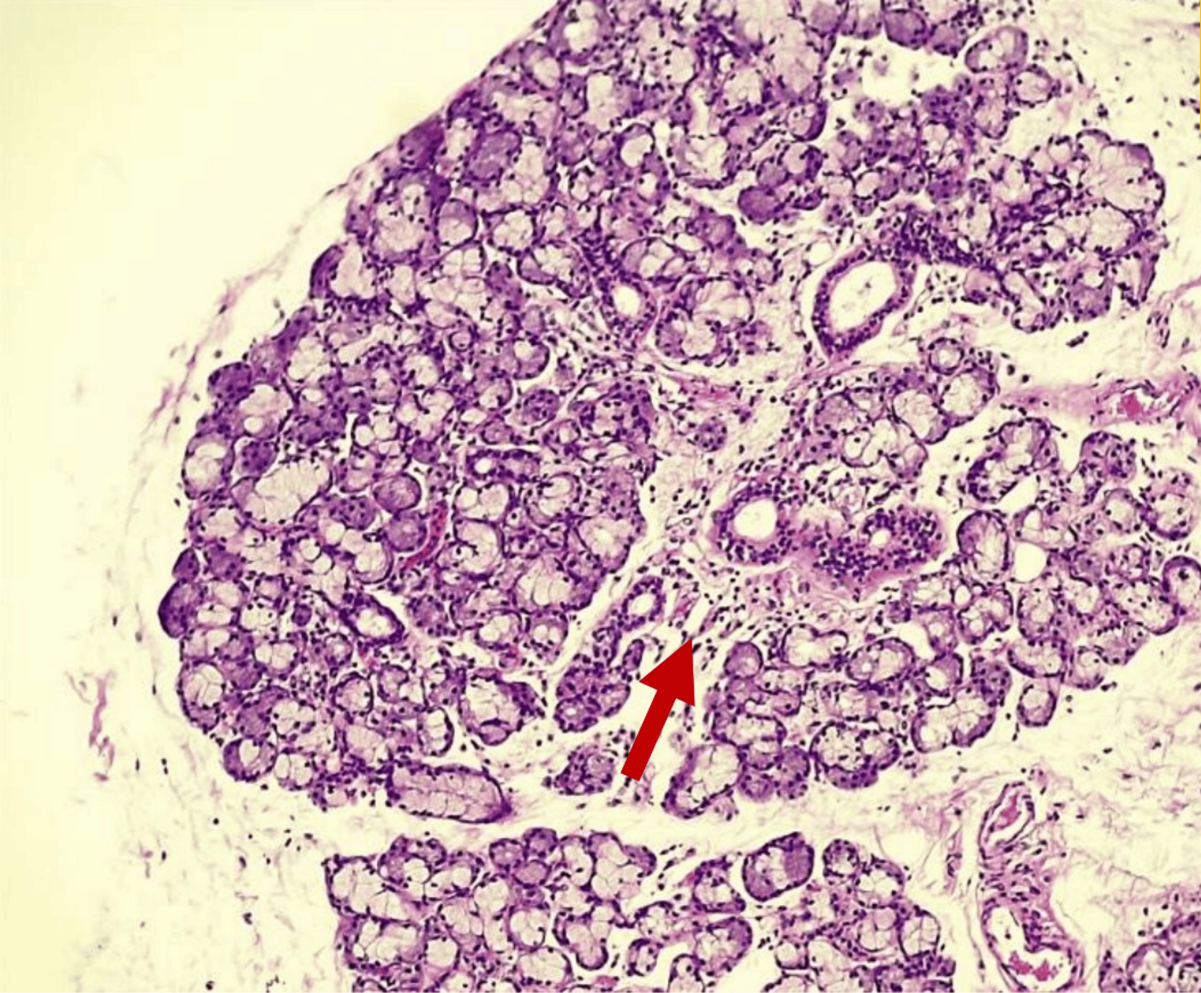
Minor salivary gland biopsy (hematoxylin and eosin stain, 100×) Higher magnification demonstrating focal lymphocytic infiltration involving the periductal and interstitial areas, associated with acinar atrophy and ductal dilatation

**Figure 3:**
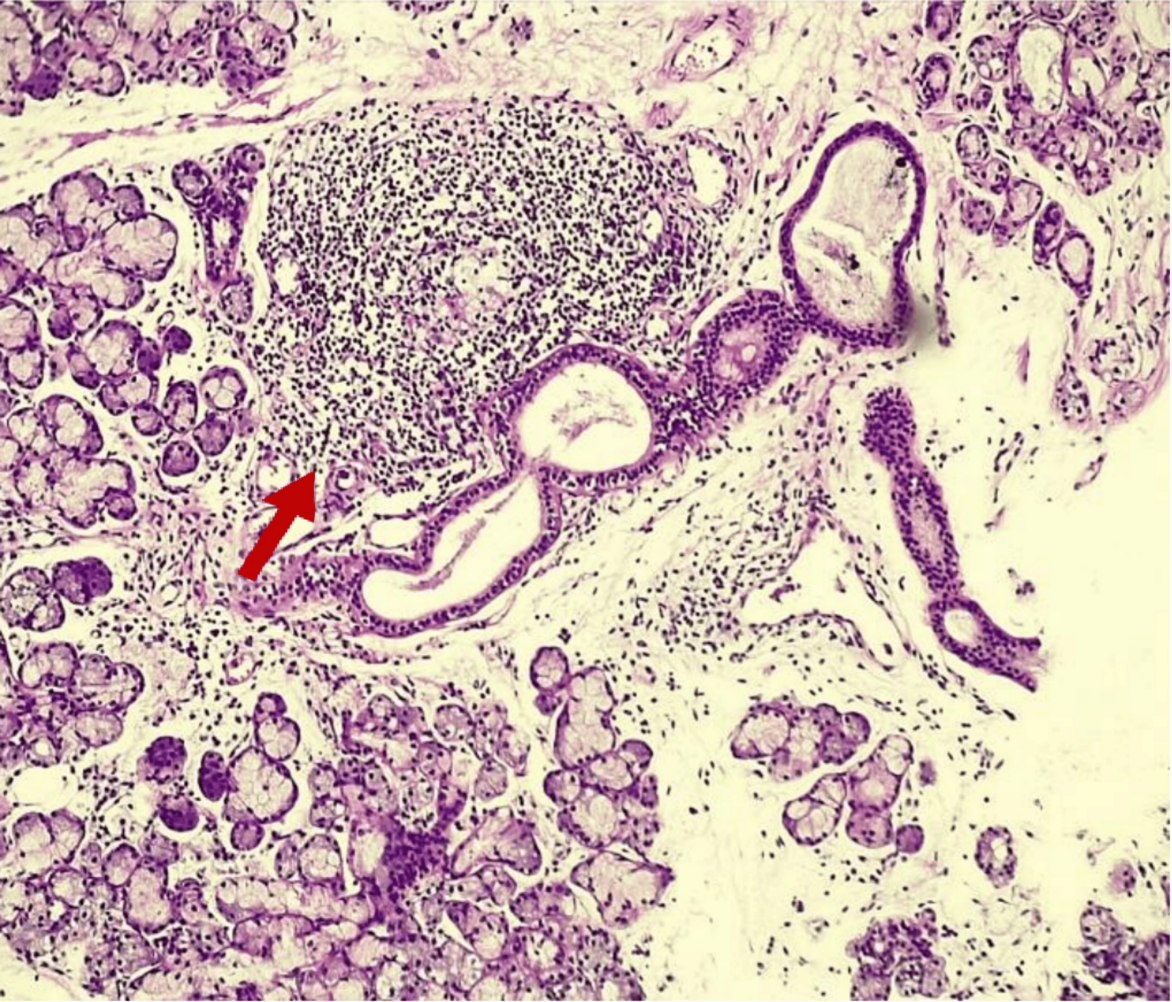
Minor salivary gland biopsy (hematoxylin and eosin stain, 100×) Dense lymphocytic aggregate consistent with a focus lesion (arrow), adjacent to salivary ducts and acini, supporting the diagnosis of multifocal lymphocytic sialadenitis with a focus score compatible with Sjögren syndrome

**Figure 4:**
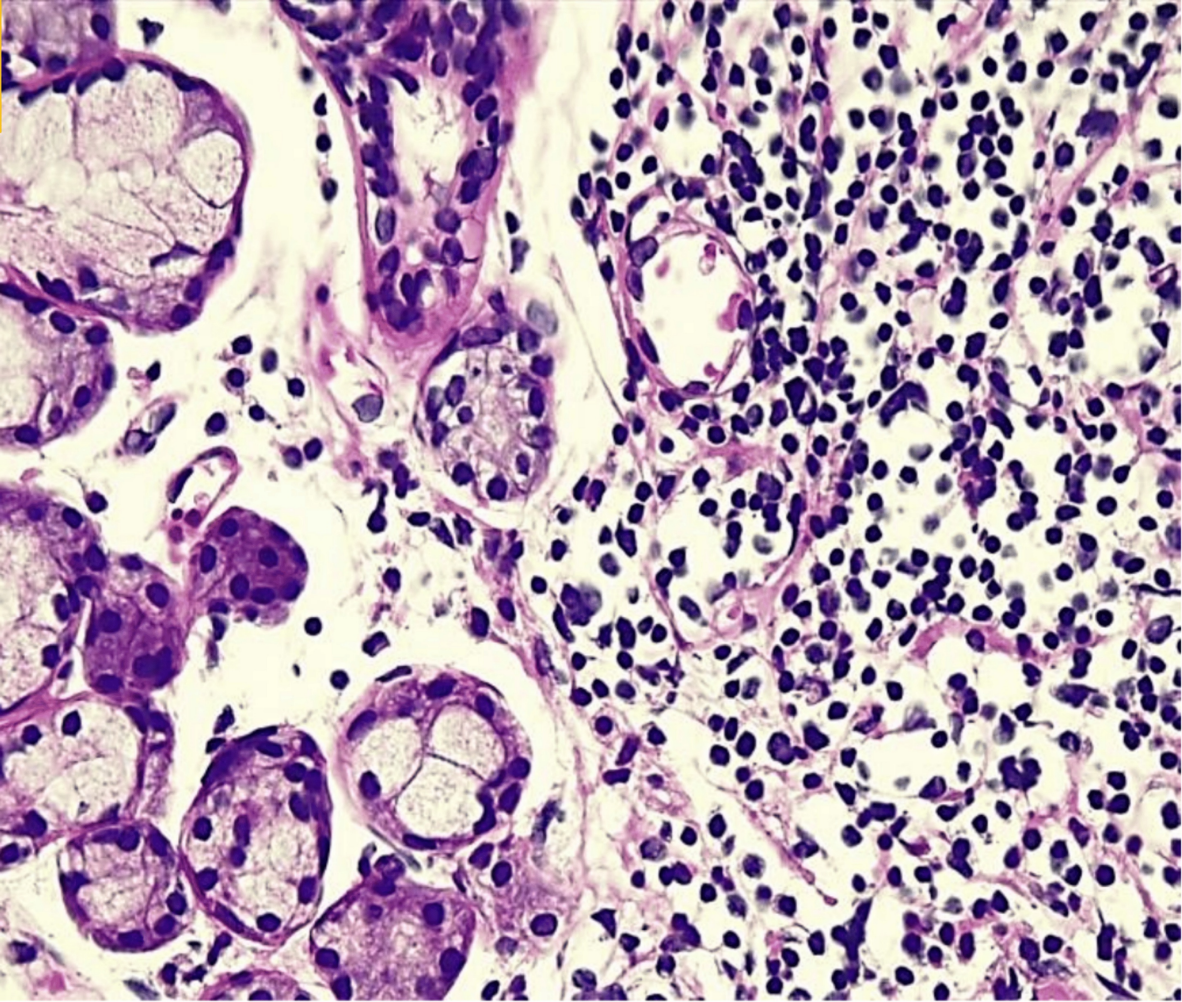
Minor salivary gland biopsy (hematoxylin and eosin, 400×) High-power view showing a dense focal lymphocytic infiltrate composed of an aggregate of 50 or more lymphocytes, consistent with a focus lesion, adjacent to salivary acini and ducts. This finding supports the diagnosis of multifocal lymphocytic sialadenitis in the setting of Sjögren syndrome

A minor salivary gland biopsy demonstrated multifocal lymphocytic sialadenitis, mild salivary gland atrophy, ductal dilatation, and mild parenchymal fibrosis, with a focus score consistent with Sjögren syndrome (Figures 1-4).

Renal biopsy revealed a fragment of renal parenchyma containing 11 glomeruli per section. Eight glomeruli (72.7%) demonstrated global sclerosis or were in advanced stages of sclerosis. Among the remaining glomeruli, two (18.2%) showed segmental sclerotic lesions with cicatricial features and synechiae formation, as well as fibrous crescent formation. The remaining glomerulus showed residual viable capillary loops without active proliferative lesions. In addition, chronic tubulointerstitial nephritis, multifocal acute tubular injury, extensive interstitial fibrosis involving more than 50% of the interstitium, and chronic arteriopathy were identified. Immunofluorescence studies were negative for immune complex deposition. Overall, these findings were consistent with pauci-immune crescentic GN with advanced chronic changes.

The diagnosis of primary Sjögren syndrome was established according to the 2016 ACR/EULAR classification criteria. The patient fulfilled the criteria through positive anti-Sjögren syndrome-related antigen A (anti-SSA/Ro) antibodies (3 points) and a minor salivary gland biopsy demonstrating focal lymphocytic sialadenitis with a focus score ≥1 focus per 4 mm² (3 points), reaching the required threshold for classification.

The patient received intravenous methylprednisolone pulse therapy at a dose of 1 g once daily for three consecutive days. This intervention was followed by a gradual improvement in renal function and proteinuria. Twenty-four-hour urinary protein excretion decreased to 2.2 g within three days and further declined to 0.66 g after four months of follow-up. Serum creatinine levels also improved, reaching 1.1 mg/dL, and the patient continues with regular outpatient follow-up.

## Discussion

SS is a systemic autoimmune disease that may present with multisystem involvement, less frequently affecting organs such as the kidneys, lymph nodes, muscles, and the central nervous system [6]. From an immunological perspective, both innate and adaptive immune responses play a significant role in the initiation and perpetuation of the disease. In glandular lesions, T lymphocytes, particularly CD4+ cells, predominate in the early stages, whereas B lymphocytes become more prominent in advanced disease [5,6]. T helper cells differentiate into subpopulations, including Th1, Th2, Th17, and T follicular helper cells, each characterized by specific cytokine profiles that contribute to the pathogenesis of SS [6,7]. In this context, Th1-type immune responses predominate within inflammatory infiltrates, although contributions from Th2 and Th17 pathways have also been described [6].

GN encompasses a heterogeneous group of immune-mediated disorders characterized by glomerular inflammation. Its clinical presentation is variable, ranging from asymptomatic hematuria and proteinuria to severe forms such as acute nephritic syndrome or rapidly progressive GN, which are more frequently observed in conditions such as postinfectious GN, ANCA-associated vasculitis, and anti-glomerular basement membrane disease [8]. From an etiological standpoint, GN may be classified into infection-related, autoimmune, alloimmune, autoinflammatory, and monoclonal gammopathy-associated forms [9,10]. Histopathologically, GN is characterized by increased glomerular cellularity resulting from intrinsic cell proliferation and/or leukocyte infiltration [10]. Renal biopsy remains the gold standard for diagnosis, allowing direct assessment of glomerular inflammation and identification of specific histopathological patterns that are essential for classification and prognostication [11]. The pathogenesis of GN involves complex interactions between bone marrow-derived inflammatory cells and resident renal parenchymal cells, with immune-mediated injury playing a central role in disease development [12,13].

Renal involvement in SS is a recognized extraglandular manifestation, although it is less common than other systemic complications. Tubulointerstitial nephritis represents the most frequent form of renal involvement, whereas GN occurs less often [16]. When glomerular involvement is present, it is typically mediated by immune complexes, with cryoglobulinemic GN being the most representative entity [16]. This form of GN is characterized by the presence of cryoglobulins, immunoglobulins that precipitate at low temperatures and induce glomerular inflammation and structural damage upon deposition within the glomeruli [16].

From a histological perspective, renal findings in patients with SS indicate that tubulointerstitial nephritis occurs in approximately 71% of cases, followed by cryoglobulinemic GN in 8%, membranous GN in 4%, and global glomerulosclerosis in 4% [17]. Cryoglobulinemic GN typically presents as membranoproliferative GN on renal biopsy and may be associated with systemic vasculitis, often requiring aggressive immunosuppressive regimens, including glucocorticoids, rituximab, and, in selected cases, plasma exchange [16,17]. In contrast, pauci-immune GN associated with SS represents an exceptional entity. This histopathological pattern, characterized by the absence or paucity of immune deposits on immunofluorescence, is usually associated with ANCA-related vasculitis; however, in the context of SS, it may occur even in the absence of these antibodies, as observed in the present case. This atypical presentation highlights the heterogeneity of the immunopathological mechanisms involved in renal injury associated with SS [16,17].

Treatment of pauci-immune GN in patients with SS generally follows therapeutic strategies used for ANCA-associated vasculitis, including glucocorticoids and immunosuppressive agents such as cyclophosphamide or rituximab [17]. Although treatment response is often favorable, prognosis largely depends on the severity of renal involvement at the time of diagnosis and on the presence of advanced interstitial fibrosis [17]. Pauci-immune crescentic GN represents an exceptionally rare renal manifestation of primary SS. To date, only a limited number of cases have been reported in the literature, mostly as isolated case reports or small case series. In several published cases, pauci-immune GN occurred in association with antineutrophil cytoplasmic antibody positivity, suggesting overlap with ANCA-associated vasculitis [17,18]. However, ANCA-negative presentations have been reported only sporadically. A pediatric case of ANCA-negative pauci-immune crescentic GN in primary SS was described by Kagan et al., highlighting that this entity can occur independently of ANCA positivity [19].

Other reports have described adult patients with ANCA-positive pauci-immune GN in the context of primary SS [20]. Compared with previously reported cases, the present case is distinctive due to the patient’s young adult age, ANCA-negative serology, rapidly progressive renal dysfunction as the initial manifestation, and histopathological evidence of advanced chronic changes. These findings expand the clinical spectrum of renal involvement in primary SS and underscore the importance of considering pauci-immune GN even in the absence of antineutrophil cytoplasmic antibodies. Although the association between SS and pauci-immune GN is uncommon, it should be considered in patients with SS who present with rapidly progressive renal dysfunction or atypical renal manifestations. Early recognition of this entity and timely initiation of immunosuppressive therapy may significantly influence clinical outcomes and preservation of renal function.

## Conclusions

Pauci-immune GN represents an uncommon renal manifestation of primary SS and may occur in the absence of ANCAs. This report highlights the importance of maintaining a high index of suspicion for atypical forms of renal involvement in patients with SS presenting with rapidly progressive renal dysfunction. Timely renal biopsy enabled definitive histopathological diagnosis and early initiation of immunosuppressive therapy, which was associated with improved renal function in our patient. Although causal relationships and treatment outcomes cannot be generalized from a single case, this report underscores the heterogeneity of renal manifestations in SS and supports an individualized approach to diagnosis and management.
